# A neural network computational structure for the fractional order breast cancer model

**DOI:** 10.1038/s41598-023-50045-z

**Published:** 2023-12-20

**Authors:** Zhenglin Huang, Qusain Haider, Zulqurnain Sabir, Mubashar Arshad, Bushra Khatoon Siddiqui, Mohammad Mahtab Alam

**Affiliations:** 1 North China Institute of Computing Technology, Beijing, 100000 China; 2 Department of Mathematics, University of Gujrat, Gujrat, 50700 Pakistan; 3 Institute for Numerical and Applied Mathematics, University of Göttingen, 37083 Göttingen, Germany; 4 Department of Computer Science and Mathematics, Lebanese American University, Beirut, Lebanon; 5 Department of Mathematics, Abbotabad University Science and Technology, Abbottabad, 22500 Pakistan; 6 Department of Mathematics, COMSATS University Islamabad, Wah Campus, Wah Cantt, 47040 Pakistan; 7 Department of Basic Medical Sciences, College of Applied Medical Science, King Khalid University, 61421 Abha, Saudi Arabia

**Keywords:** Fungal infection, Biomedical materials

## Abstract

The current study provides the numerical performances of the fractional kind of breast cancer (FKBC) model, which are based on five different classes including cancer stem cells, healthy cells, tumor cells, excess estrogen, and immune cells. The motive to introduce the fractional order derivatives is to present more precise solutions as compared to integer order. A stochastic computing reliable scheme based on the Levenberg Marquardt backpropagation neural networks (LMBNNS) is proposed to solve three different cases of the fractional order values of the FKBC model. A designed dataset is constructed by using the Adam solver in order to reduce the mean square error by taking the data performances as 9% for both testing and validation, while 82% is used for training. The correctness of the solver is approved through the negligible absolute error and matching of the solutions for each model’s case. To validates the accuracy, and consistency of the solver, the performances based on the error histogram, transition state, and regression for solving the FKBC model.

## Introduction

There are various dangerous diseases among them breast cancer (BC) is one of the serious global health issues, which is commonly found in the women throughout the world. The breast's epidermal cells are the first to become cancerous tumors, which can then spread to other parts of the human organism or attack tissues that are nearby. BC can develop in the breast’s lobes or in the inner ducts layer that produce milk, which is documented as lobular carcinoma or ductal carcinoma^1^. Several mathematical frameworks have been created over the investigations of BC to learn more about how it develops and potential treatments. This cancer also occurred in male with rare cases with the rate of occurrence of 0.5% to 1% as compared to female BC^2–5^.

BC is considered more complicated in women as compared to men and the chances of recovery in men are high in comparison with the females^6^. The detailed study based on the connection of male and female BC is presented by Miao^7^. Those women who are married with close relatives can have higher chances of BC. Couch^8^ highlighted the importance of breast cancer based on the susceptibility genes using the large triple-negative BC cohort unselected based on the family history of BC. It is the second most invasive cancer worldwide that wrapped around 2 million new people into positive cases each year. This high number takes into the dangerous zone, which is required to have a need for better preventative measures, early diagnosis techniques, and cutting-edge therapeutic approaches. BC produces various risk factors including those connected to reproduction, hormones, obesity, and a favorable family history. The difference between early and late stages of the BC is dependent on the age-specific incidence rates^9^. Fan et al.^10^ discussed the mathematical models using the bioinformatical and computational studies, which help to monitor the damage of Deoxyribonucleic acid recognition, tumor growth, chromatin remodeling, cellular distribution, and checkpoint regulator. Mufudza et al.^11^ discussed the organizational impacts of all known single nucleotide polymorphisms or genetic variants in the breast cancer genes. The initial stage of BC creates the physical irregularity can be controlled by using the good treatment and early detection is crucial based on the effective measures of the disease. Bray et al.^12^ provided the several risk factors including the reproduction, hormones, obesity, and a favorable family history with the role in BC development. Arshad et al.^13^ described the pathogenic mutations with a high propensity to produce cancer located areas of the gene.

There are various studies that highlights the importance of genetic profiling based on BC prevention and its therapy by illuminating the role of genes using a predisposing individual of the illness^1,14,15^. Most pathogenic mutations with a high propensity to cause cancer are areas of the gene. Genes play a key role in the development of BC using the increase of estrogen level, which causes a natural body response toward growing the tumors in the BC dynamics^16^. Enderling et al.^17^ presented the mathematical cancerous cell model using the regulated healthy pre-cancerous cells formed in the post-pubertal BC structure. BC development with higher estrogen levels is studied with the impacts of healthy breast epithelial cells, interactions among cancer cells, the body’s natural immune response, and non – proliferative tumor cells^18^.

Mathematical systems are important tools in numerous fields of engineering, science, and economics. There are several mathematical models that have been studied in current decades and the solution of these models have been presented by using different schemes. To mention a few of them, Ganji et al.^19^ presented the solutions of the brain tumor model using the fractional operator. Sánchez et al.^20^ provided the solutions of the coronavirus model by applying a reliable numerical scheme. Sabir et al.^21^ provided the results of prediction differential system by using the explicit Runge–Kutta and Adams numerical techniques. Hart et al.^22^ discussed the mathematical modelling of the functionally dependent bone remodeling and provided its numerical solutions. Fogelson et al.^23^ designed and studied numerically by using a mathematical system based on the platelet adhesion and aggregation during blood clotting. Sana et al.^24^ discussed a mathematical model based on the supply chain systems. The coronavirus time series data by applying the spectral analysis and deep learning methods have been proposed by Oshinubi et al.^25^. Few recent mathematical models that have been used to solve with different numerical schemes are provided in these references^26–28^.

The purpose of this research is to obtain the solutions of the fractional kind of breast cancer (FKBC) system in order to get more precise solutions as compared to integer order. Hence, a reliable stochastic scheme based on the Levenberg Marquardt backpropagation neural networks (LMBNNS) is proposed for the FKBC model. The stochastic process has been derived before to solve various kind of the mathematical model, but the solutions of the FKBC model have been presented first time with the implementation of this scheme. Currently, the neural network-based procedures have been used to solve functional form of singular models^29^, hepatitis virus models^30^, bone remodeling model^31^, fractional vector-host diseased model^32^, SIRC epidemic model^33^, Zika virus model^34^, breathing transmission system^35^, thermal explosion model^36^, Rabinovich–Fabrikant system^37^, Layla and Majnun model^38^ and food chain model^39^.

The fractional order models are considered more challenging and provide more reliable results to solve the differential model. Fractional types of derivatives are implemented to test the effectiveness of the real-world applications^16,17^. Over the past 3 decades, the implementation of fractional calculus has been observed widely by applying the powerful operators of Weyl-Riesz^18^, Caputo^19^, Riemann–Liouville^20^, Erdlyi-Kober^21^, and Grnwald-Letnikov^22^. The Caputo derivative can be applied to solve both conditions of the model homogeneous and non-homogeneous. All these operators have their own drawbacks; however, the Caputo derivatives are considered simple as compared to other operators. Some novel motivations of this study are given as:

- The design of the FKBC system have been provided and the solutions have been performed by using the LMBNNs.
- Three different cases based on the fractional order have been presented to solve the FKBC system.
- The correctness of the scheme is observed by using the overlapping of the outputs.
- The absolute error (AE) results in good performances enhance the reliability of the proposed solver.

The other paper's parts are organized as: Sect. "Mathematical FKBC system" describes the construction of the FKBC system, Sect. "Designed LMBNNs procedure" provides the proposed technique based LMBNNs, Sect. "Results and discussions" is constructed based on the calculated outcomes, while conclusions are proposed in Sect. "Conclusions".

## Mathematical FKBC system

This section provides the FKBC system, which is divided into five different categories named as cancer stem cells $C(t)$, tumor cells $T(t)$, healthy cells $H(t)$, immune cells $I(t)$, and excess estrogen $E(t)$. The mathematical form of the integer order BC model is given as^16^:

1 $$\left\{ \begin{gathered} \frac{dC(t)}{{dt}} = k_{1} C(t)\left( {1 - \frac{C(t)}{{M_{1} }}} \right) - \gamma_{1} C(t)I(t) + \frac{{p_{1} E(t)C(t)}}{{a_{1} + C(t)}}, \hfill \\ \frac{dT(t)}{{dt}} = k_{2} C(t)\left( {\frac{C(t)}{{M_{1} }}} \right)\left( {1 - \frac{T(t)}{{M_{2} }}} \right) - n_{1} T(t) - \gamma_{2} T(t)I(t) + \frac{{p_{2} T(t)E(t)}}{{a_{2} + T(t)}}, \hfill \\ \frac{dH(t)}{{dt}} = q\left( {1 - \frac{H(t)}{{M_{3} }}} \right)H(t) - \delta T(t)H(t) - \frac{{p_{3} E(t)H(t)}}{{a_{3} + H(t)}}, \hfill \\ \frac{dI(t)}{{dt}} = s + \frac{\rho I(t)T(t)}{{T(t) + w}} - \gamma_{3} T(t)I(t) - n_{2} I(t) - \frac{uE(t)I(t)}{{E(t) + \upsilon }}, \hfill \\ \frac{dE(t)}{{dt}} = \tau - \left( {\mu + \frac{{d_{1} C(t)}}{{C(t) + a_{1} }} + \frac{{d_{2} T(t)}}{{T(t) + a_{2} }} + \frac{{d_{3} H(t)}}{{H(t) + a_{3} }}} \right), \hfill \\ \end{gathered} \right.$$

where $k_{1}$, $k_{2}$ and $q$ denote the normal rate of cell separation for first three dynamics, and *M*_1_ indicates the carrying size based on the cells of first three dynamics. The rates at which estrogen promotes the growth of cancer stem and tumor cells, as well as the rates at which healthy cells are lost because of estrogen-induced DNA mutation, are shown in *p*_1_, *p*_2_, and *p*_3_, respectively. It is also presented the rates at which immune cells react to tumor cells and cancer stem cells; *a*_1_, *a*_2_, and *a*_3_ designate the number of first three dynamics. The values of *n*_1_ and *n*_2_ are the usual tumor. In addition, the rate at which healthy cells decease, *s* also shows the basis rate of resistant cells. *u* is the rate of immunological suppression caused by estrogen; *v* is the immune cell threshold; The letters $\tau$ stands the continuous estrogen infusion, the body's estrogen washout rate, and the letters *d*_1_, *d*_2_ and *d*_3_ are the rates at which cancer stem cells, tumor cells, and healthy cells absorb estrogen, respectively. The fractional form of the above system becomes as Ref.^16^:

2 $$\left\{ \begin{gathered} \frac{{d^{\alpha } C(t)}}{{dt^{\alpha } }} = k_{1} C(t)\left( {1 - \frac{C(t)}{{M_{1} }}} \right) - \gamma_{1} C(t)I(t) + \frac{{p_{1} E(t)C(t)}}{{a_{1} + C(t)}}, \hfill \\ \frac{{d^{\alpha } T(t)}}{{dt^{\alpha } }} = k_{2} C(t)\left( {\frac{C(t)}{{M_{1} }}} \right)\left( {1 - \frac{T(t)}{{M_{2} }}} \right) - n_{1} T(t) - \gamma_{2} T(t)I(t) + \frac{{p_{2} E(t)T(t)}}{{a_{2} + T(t)}}, \hfill \\ \frac{{d^{\alpha } H(t)}}{{dt^{\alpha } }} = qH(t)\left( {1 - \frac{H(t)}{{M_{3} }}} \right) - \delta T(t)H(t) - \frac{{p_{3} E(t)H(t)}}{{a_{3} + H(t)}}, \hfill \\ \frac{{d^{\alpha } I(t)}}{{dt^{\alpha } }} = s + \frac{\rho I(t)T(t)}{{w + T(t)}} - \gamma_{3} T(t)I(t) - n_{2} I(t) - \frac{uE(t)I(t)}{{\upsilon + E(t)}}, \hfill \\ \frac{{d^{\alpha } E(t)}}{{dt^{\alpha } }} = \tau - \left( {\mu + \frac{{d_{1} C(t)}}{{a_{1} + C(t)}} + \frac{{d_{2} T(t)}}{{a_{2} + T(t)}} + \frac{{d_{3} H(t)}}{{a_{3} + H(t)}}} \right), \hfill \\ \end{gathered} \right.$$

where $\alpha$ stands the fractional order Caputo derivative. To investigate the complicated features, such as super slow evolution and superfast transients are considered challenging as compared to integer order given in system (1). There are various recent applications, where the fractional kind of the derivatives have been used. Some of them are inconsistent heat transmission^40^, pine wilt disease model with convex rate^41^, spatiotemporal outlines^42^, and soil animal approximation^43^, and soil animal constituent content^44^.

## Designed LMBNNs procedure

**Figure 1:**
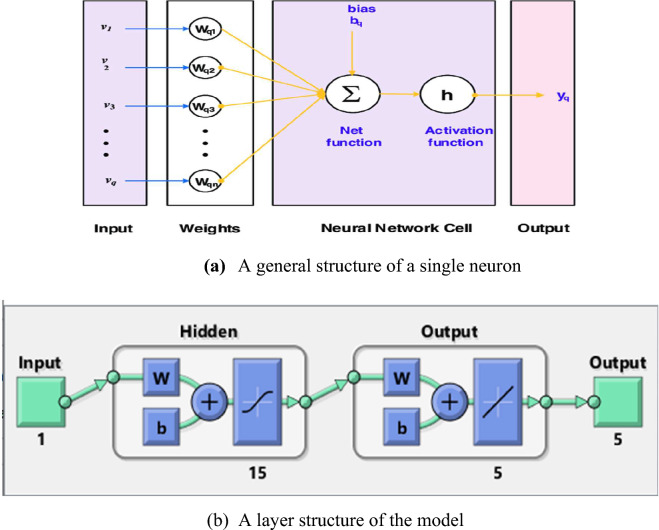
(**a**) A general structure of a single neuron. (**b**) A layer structure of the model.

The stochastic computational performances based on the LMBNNs scheme to solve the mathematical FKBC system as stated in the set of systems (2) is provided in this section. Three components of model using the developed technique, and outcomes are provided in Fig. 1a and b. These Figs depict the workflow diagram for the FKBC system. The design presentations are described in two different metrics including LMBNNs-based processes and the mathematical process. The design of the dataset is presented by using the Adam scheme and the division of the data contains 82% (training) and 9% for both verification and testing by taking 15 hidden neurons. Log-sigmoid transfer function in the hidden layers is selected and single input and output layer structure based on the neurons is obtained. The current neural network study is operated with untimely conjunction, overfitting, and concealed scenarios. Therefore, the parameters of the networks have been used carefully after considerable tests, experience, and knowledge. A fully concentration is required to adjust the parameter setting and a small modification in the setting can change the whole scenario and can impact the performance of the investigations. The "MATLAB" software (NFTOOL command) is used to implement the stochastic process based on the LMBNNs, which include the correct hidden neuron sections, testing statistics, learning strategies, and verification statics. Table 1 shows the parameter setting to perform the LMBNNs for solving the FKBC model.

**Table 1:** Parameters adjustment to perform the LMBNNs.

Index	Settings
Activation function	Log-sigmoid
Fitness goal (MSE)	0
Minimum gradient	10^–07^
Maximum Mu values	10^10^
Hidden neurons	15
Maximum Epochs	1000
Output layer	Single
Increasing Mu performances	10
Validation statics	9%
Authentication fail amount	6
Lower Mu values	0.1
Sample assortment	Arbitrary
Training data	82%
Adaptive parameter	7 × 10^–05^
Testing data	9%
Input layer	Single
Dataset	Adam technique
Adam solver implementations	Default
Stoppage criteria	Default

## Results and discussions

In this section, three different cases have been taken by using the values of $\alpha =0.5, 0.7\, \mathrm{and\, }0.9$ for solving the mathematical model.

### Analysis of results for case 1

Consider the values $\alpha =0.5$, ${k}_{1}=0.05$, ${M}_{1}=0.02$, ${p}_{1}=0.005$, ${a}_{1}=0.6$, ${\gamma }_{1}=0.01$, ${p}_{2}=0.01$, ${a}_{2}=0.07$, ${k}_{2}=0.02$, ${M}_{2}=0.5$, ${\gamma }_{2}=0.1$, ${n}_{1}=0.3$, $q=0.3$, ${M}_{3}=0.4$, $p_{3} = 0.5$ ${a}_{3}=0.5$, $\delta =0.05$, $s=0.02$, ${n}_{2}=0.005$, ${\gamma }_{3}=0.6$, $u=0.01$, $v=0.01$, $\rho =0.07$, $\omega =0.02$, $\tau =0.5$, $\mu =0.1$, ${d}_{1}=0.3$, ${d}_{2}=0.3$, ${d}_{3}=0.5$ in Eq. (2), while the values of the initial conditions have been selected 1.2 for each class.

**Figure 2:**
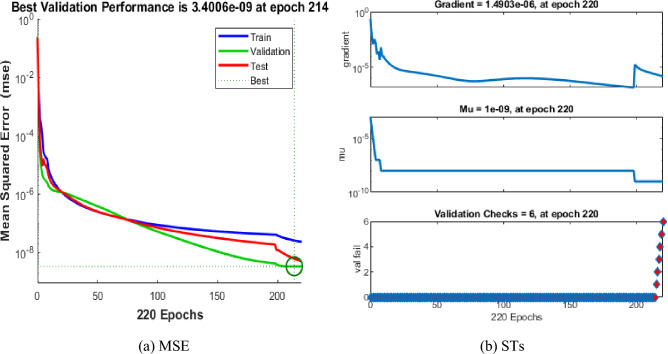
MSE and STs for the FKBC system based case 1.

**Figure 3:**
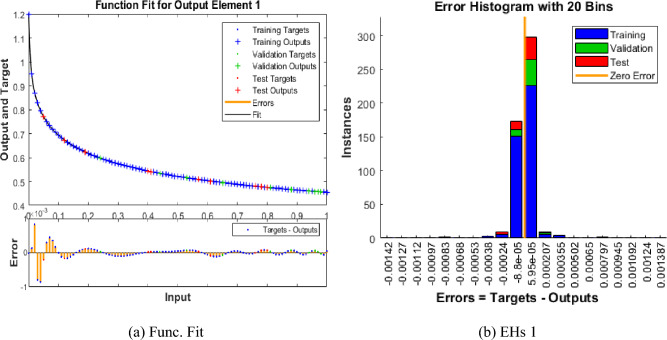
Func fit and EHs for the mathematical system for case 1.

**Figure 4:**
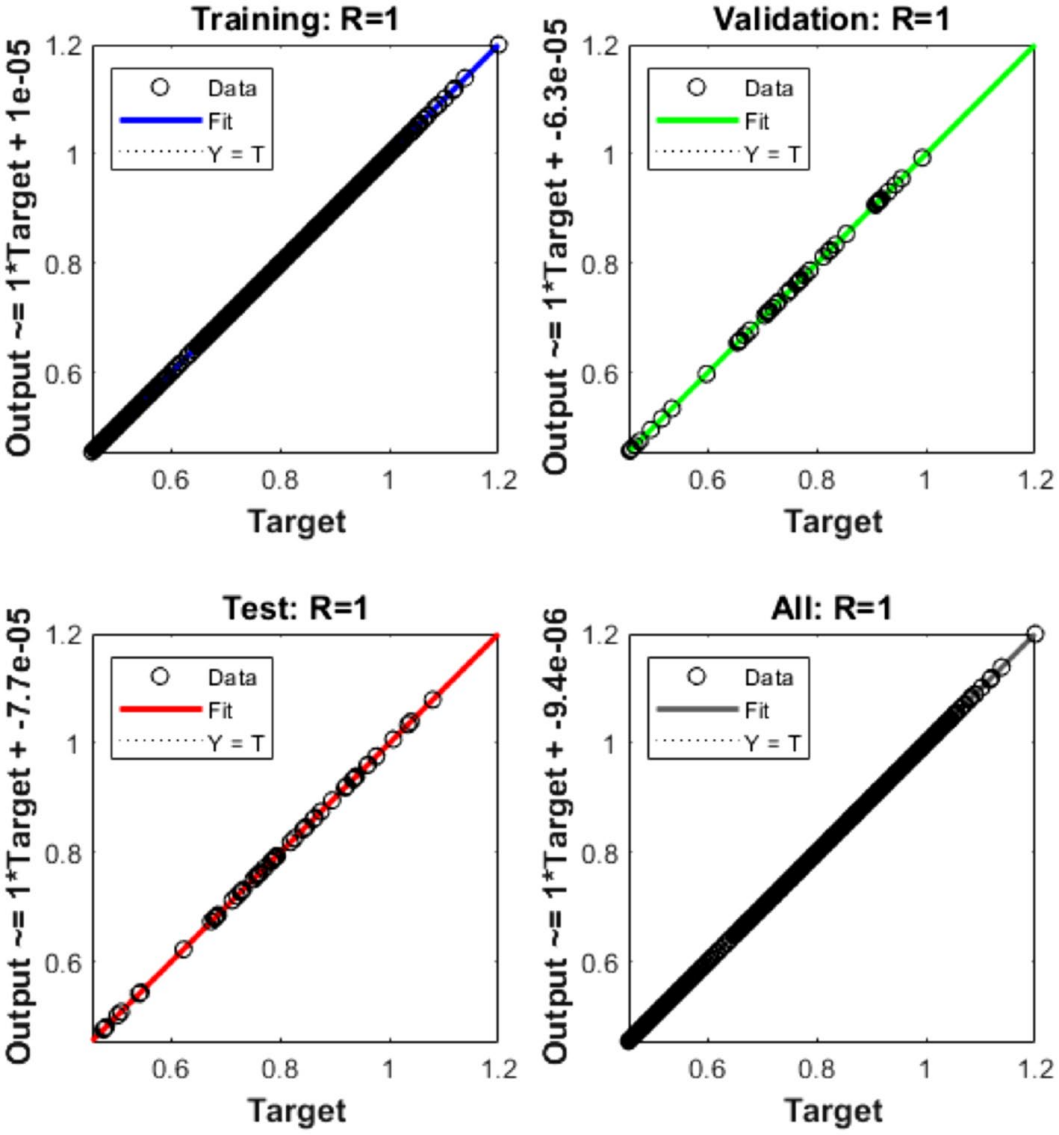
Regression for the FKBC model (1).

The mean square error (MSE) and state evolution (SE) results of the FKBC model's performance are shown in Fig. 2a,b for case 1. The first two parts of the Fig. 2 indicate the decrement of MSE for solving the FKBC model. The best validation performances are reported as 3.4006 $\times 1{0}^{-09}$, while the gradient is reported as 1.4903 $\times 1{0}^{-06}$, and the epochs have been calculated for this case are 220. Figure 3 shows the function fit and error histogram (EH) performances for case 1. The first half is designed based on the func fit, while the second half provides the EH. The values of the EH are presented as 5.95 $\times 1{0}^{-05}$. Figure 4 provides the regression performances for case 1, which is calculated as 1 that shows the perfect model.

### Analysis of results for case 2

Consider $\alpha =0.7$, ${k}_{1}=0.05$, ${M}_{1}=0.02$, ${p}_{1}=0.005$, ${a}_{1}=0.6$, ${\gamma }_{1}=0.01$, ${p}_{2}=0.01$, ${a}_{2}=0.07$, ${k}_{2}=0.02$, ${M}_{2}=0.5$, ${\gamma }_{2}=0.1$, ${n}_{1}=0.3$, $q=0.3$, ${M}_{3}=0.4$, $p_{3} = 0.5$ ${a}_{3}=0.5$, $\delta =0.05$, $s=0.02$, ${n}_{2}=0.005$, ${\gamma }_{3}=0.6$, $u=0.01$, $v=0.01$, $\rho =0.07$, $\omega =0.02$, $\tau =0.5$, $\mu =0.1$, ${d}_{1}=0.3$, ${d}_{2}=0.3$, ${d}_{3}=0.5$ in Eq. (2), while the values of the initial conditions have been selected 1.2 for each class.

**Figure 5:**
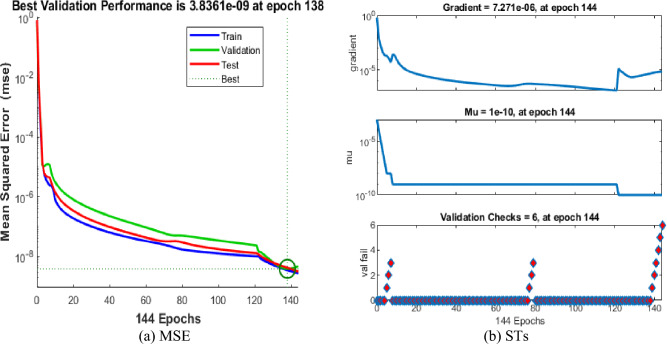
MSE and STs for the FKBC system for case 2.

**Figure 6:**
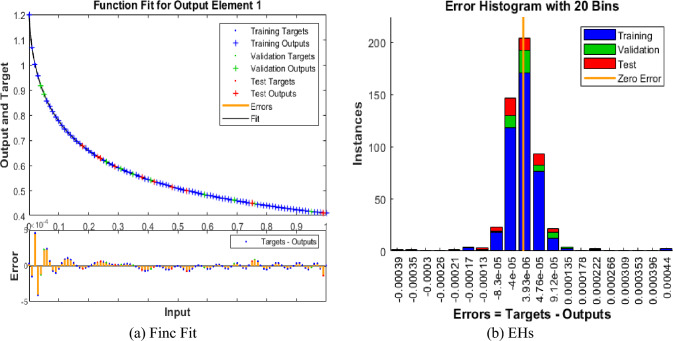
Func fit and EHs for the mathematical system based case 2.

**Figure 7:**
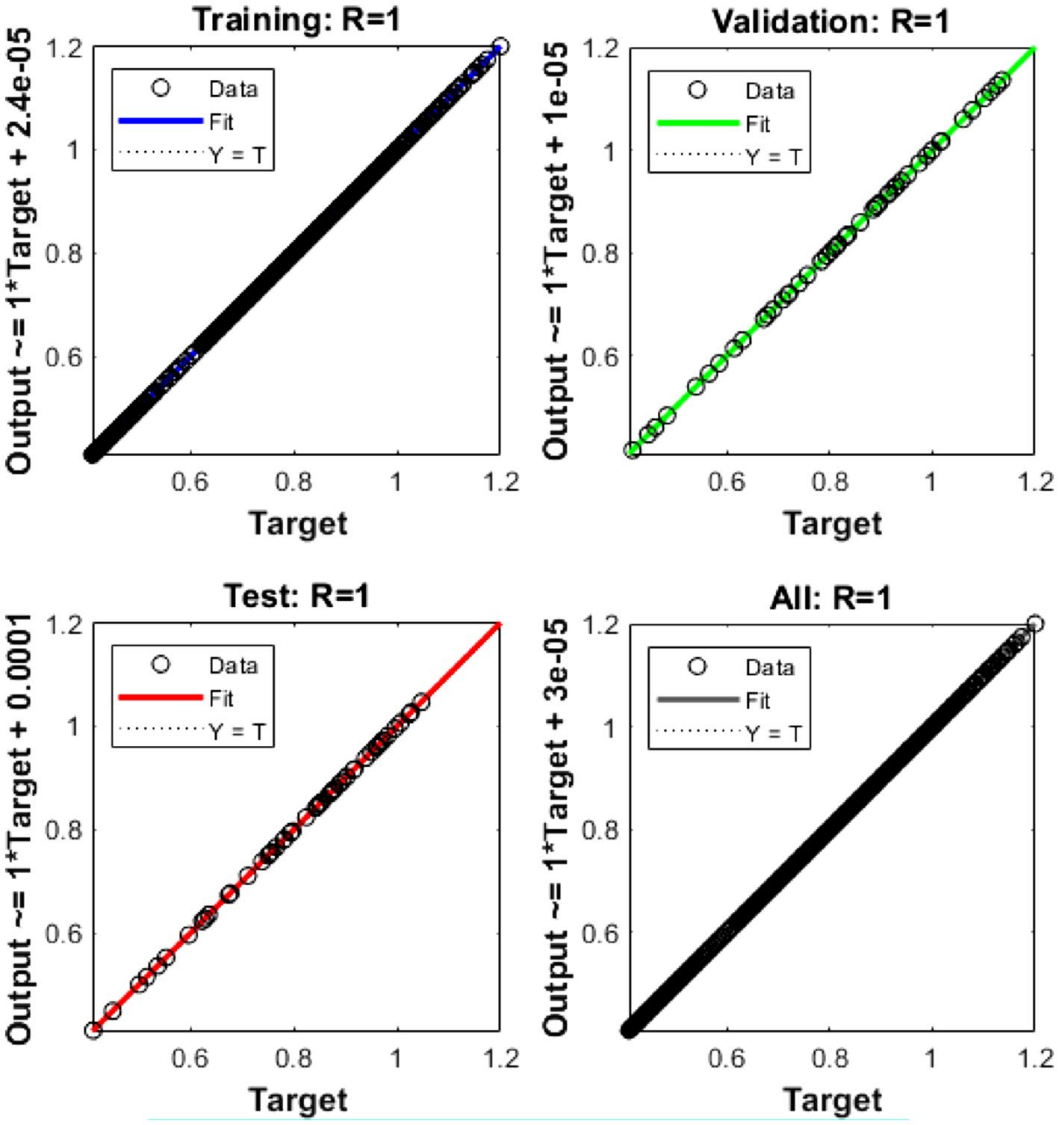
Regression for the FKBC model (2).

The values of the MSE and SE to present the FKBC model's performance are illustrated in Fig. 5a,b for case 2. The first two half of Fig. 5 represent the decrement of MSE for solving the FKBC model. The best validation performances are reported as 3.8361 $\times 1{0}^{-09}$, while the gradient is reported as 7.271 $\times 1{0}^{-06}$, and the epochs have been calculated for this case are 144. Figure 6 shows the function fit and EH performances for case 2. The first half is designed based on the func fit, while the second half provides the EH. The values of the EH are presented as 3.93 $\times 1{0}^{-06}$. Figure 7 describes the regression for case 2, which is performed as 1 that represents the perfect model.

### Analysis of results of case 3

Consider $\alpha =0.9$, ${k}_{1}=0.05$, ${M}_{1}=0.02$, ${p}_{1}=0.005$, ${a}_{1}=0.6$, ${\gamma }_{1}=0.01$, ${p}_{2}=0.01$, ${a}_{2}=0.07$, ${k}_{2}=0.02$, ${M}_{2}=0.5$, ${\gamma }_{2}=0.1$, ${n}_{1}=0.3$, $q=0.3$, ${M}_{3}=0.4$, $p_{3} = 0.5$ ${a}_{3}=0.5$, $\delta =0.05$, $s=0.02$, ${n}_{2}=0.005$, ${\gamma }_{3}=0.6$, $u=0.01$, $v=0.01$, $\rho =0.07$, $\omega =0.02$, $\tau =0.5$, $\mu =0.1$, ${d}_{1}=0.3$, ${d}_{2}=0.3$, ${d}_{3}=0.5$ in Eq. (2), while the values of the initial conditions have been selected 1.2 for each class.

**Figure 8:**
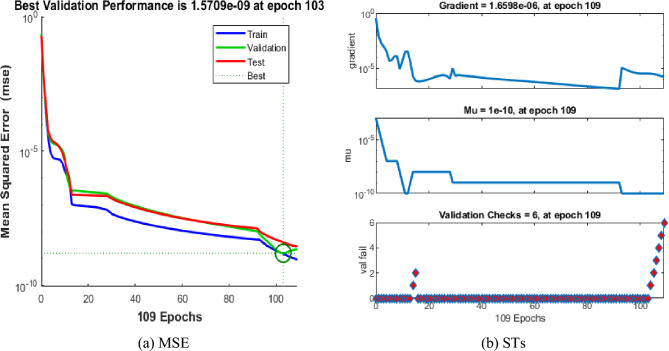
MSE and STs performances for the FKBC system for case 2.

**Figure 9:**
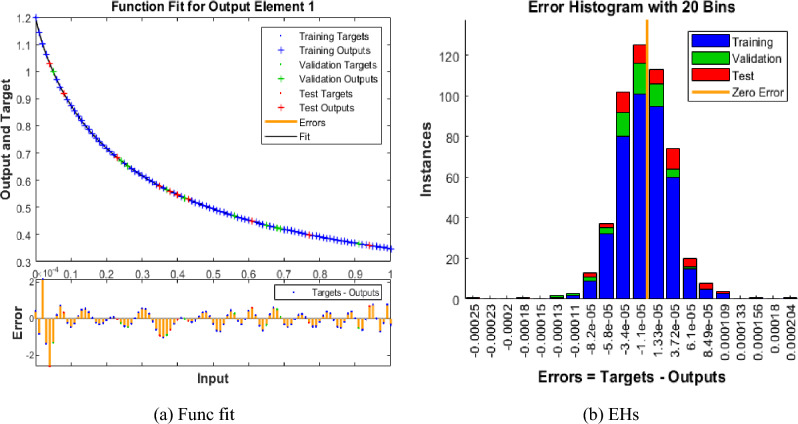
Func fit and EHs for the FKBC system for case 3.

**Figure 10:**
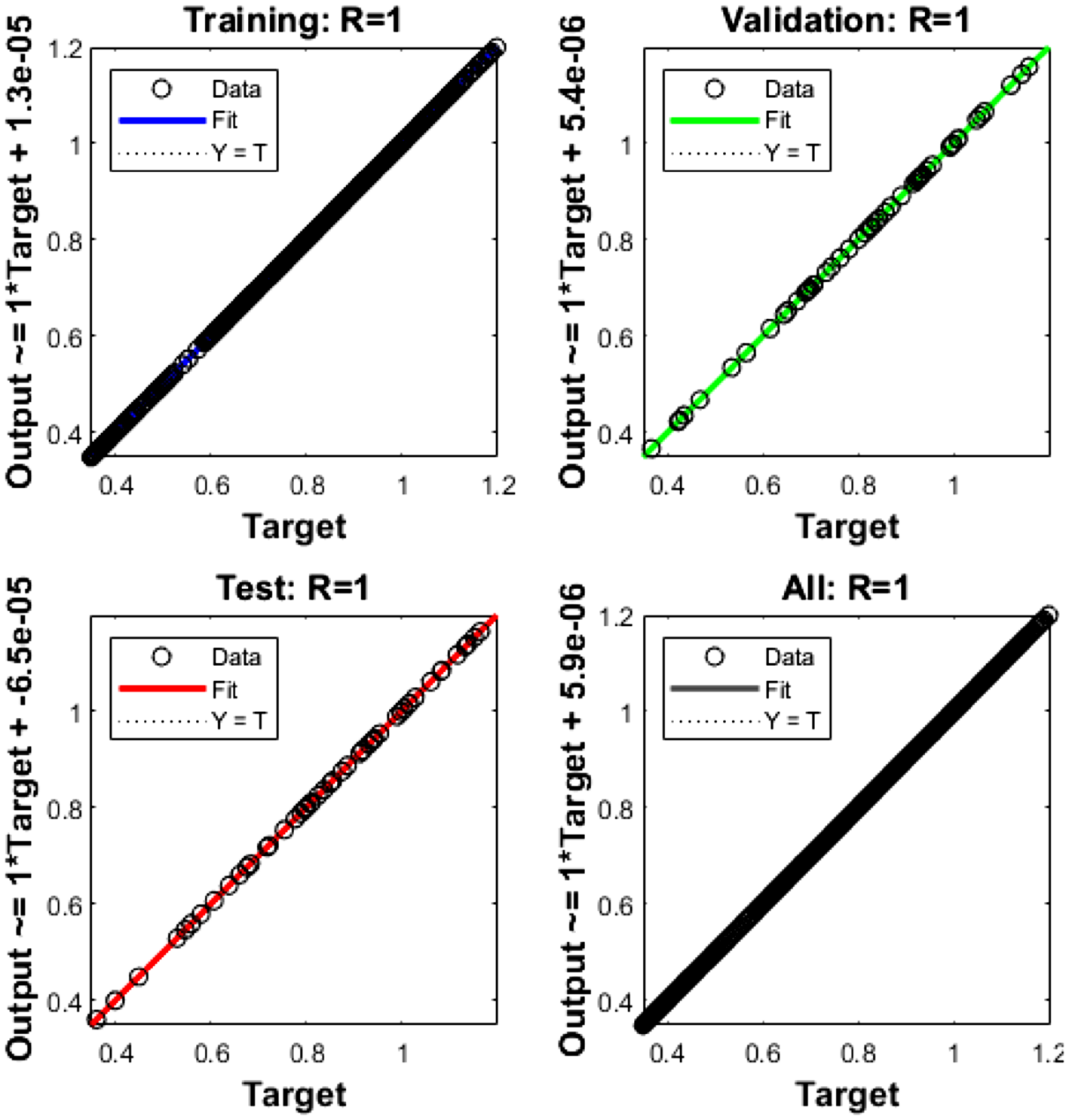
Regression for the FKBC model (3).

The values of the MSE and SE to present the FKBC model's performance are demonstrated in Fig. 8a,b for case 3. The first two half of Fig. 8 represent the decrement of MSE for solving the FKBC model. The best validation performances are reported as 1.5709 $\times 1{0}^{-09}$, while the gradient is reported as 1.6598 $\times 1{0}^{-06}$, and the epochs have been calculated for this case are 144. Figure 9 shows the function fit and EH performances for case 3. The first half is designed based on the func fit, while the second half provides the EH. The values of the EH are presented as 1.33 $\times 1{0}^{-05}$. Figure 10 describes the regression for case 3, which is performed as 1 that represents the perfect model.

**Figure 11:**
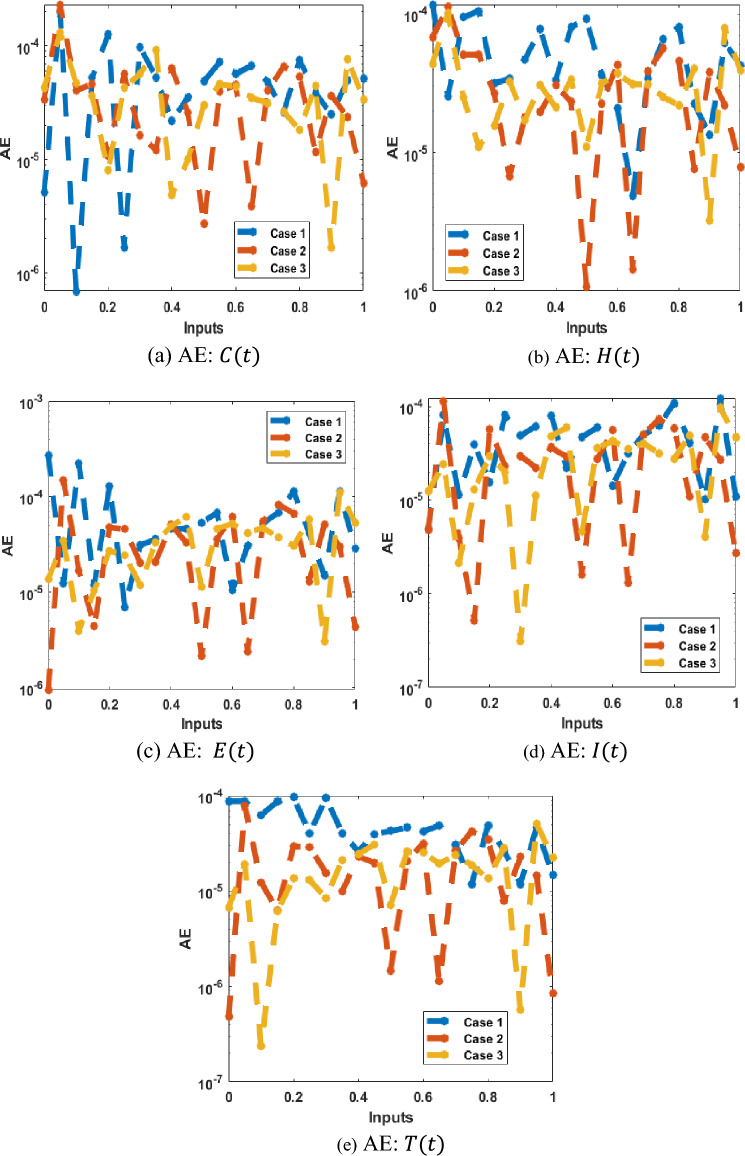
(**a**–**e**) AE values for the FKBC model.

**Figure 12:**
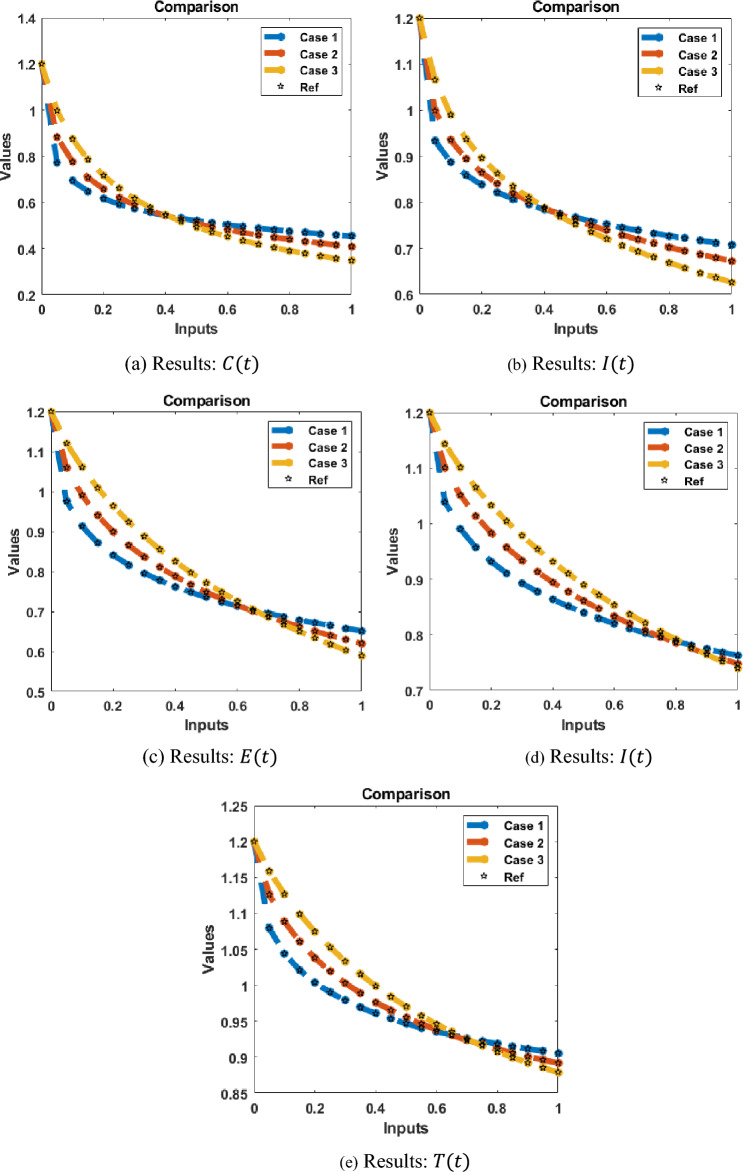
(**a**–**e**) Result assessment for the FKBC model.

The AE is reported in Fig. 11, which shows that these values are performed as 10^–04^ to 10^–06^ for each dynamic of the model. This negligible AE performance enhance the correctness of the scheme. For the solution of the FKBC model using the procedures based on the LMBNNs are presented in Fig. 12. It is reported that the matching of the results is obtained for each case of the FKBC system.

Table 2 shows the MSE performances based on the neural networks along with the counted epochs and complexity measures.

**Table 2:** Designed procedure for the FKBC model.

Case	MSE			Performance	Gradient	Mu	Epoch	Time
	[Training]	[Verification]	[Testing]					
I	2.610 × 10^−08^	3.400 × 10^−09^	6.21 × 10^−09^	2.36 × 10^−08^	1.49 × 10^−06^	1 × 10^−09^	220	01
2	3.430 × 10^−09^	3.836 × 10^−09^	4.13 × 10^−09^	2.69 × 10^−09^	7.27 × 10^−06^	1 × 10^−10^	144	01
3	1.490 × 10^−09^	1.570 × 10^−09^	3.99 × 10^−09^	9.10 × 10^−10^	1.66 × 10^−06^	1 × 10^−10^	109	01

## Conclusions

In this study, the numerical solution of the fractional breast cancer system have been presented, which are based on five different classes including cancer stem cells, healthy cells, tumor cells, excess estrogen, and immune cells. Some of the concluding remarks of this work are given as:

- The fractional derivatives have been introduced to solve the breast cancer mathematical model.
- The fractional kind of derivatives have been provided to get more precise solutions of the model as compared to integer order.
- A stochastic computing Levenberg Marquardt backpropagation neural networks scheme has been proposed for three fractional order cases of the FKBC model.
- The constructions of the designed dataset based on the Adam solver has been presented to reduce the MSE by taking the data performances as 9% for both testing and validation, while 82% is used for training.
- The correctness of the solver has been approved through the negligible absolute error along with the matching of the solutions for each model’s case.
- To authenticate the accuracy of the solver, the performances based on the error histogram, transition state, and regression for solving the FKBC model has been provided.

### Future recommendations

The designed structure based on the stochastic approach can be executed for various nonlinear natured models^45–62^.

## Data Availability

All data generated or analyzed during this study are included in this article.
